# 

**Published:** 1874-02

**Authors:** 


VOL. XXVIII. FEBRUARY, 1874. NO. 2

THE

### DENTAL REGISTER.

A MONTHLY JOURNAL

DEVOTED TO THE INTERESTS OF THE PROFESSION.

Edited by J. TAFT, D. D. S.;

AND PUBLISHED BY

SPENCER & MOORE, OHIO DENTAL DEPOT, 168 RACE ST.,
Cincinnati, Ohio.

TERMS: $2,50 PER ANNUM, IN ADVANCE; SINGLE COPIES, 25 cts

